# Supplementary material for: A UMLS-based spell checker for natural language processing in vaccine safety
Source: BMC Med Inform Decis Mak. 2007 Feb 12;7:3. doi: 10.1186/1472-6947-7-3 (PMC1805499; doi:10.1186/1472-6947-7-3)
Supplement: Additional file 13 — RAP application directory. Sets up directory for RAP (RDF application for PHP) source code [file 1472-6947-7-3-S13.gz › rap/doc/phpdoc/resModel/ResContainer.html]

xml version="1.0" encoding="iso-8859-1"?


Docs For Class ResContainer


## Class ResContainer

Description

Description |
Descendents
| Methods (details)

An RDF Container.

This Class defines methods for accessing RDF container resources. These methods operate on the RDF statements contained in a model.

History:

- access: public
- author: Daniel Westphal <at d-westphal dot de mail at d-westphal dot de>
- version: V0.9.1

Located in /resModel/ResContainer.php (line 21)

```
Object
   |
   --Node
      |
      --Resource
         |
         --ResResource
            |
            --ResContainer
```

Direct descendents

Description |
Descendents
| Methods (details)

| Class | Description |
| --- | --- |
| ResAlt | This interface defines methods for accessing RDF Alternative resources. |
| ResBag | This interface defines methods for accessing RDF Bag resources. |
| ResSeq | This interface defines methods for accessing RDF Sequence resources. |

Method Summary

Description |
Descendents |
Methods (details)

ResContainer
ResContainer
([string $uri = null])

void
add
(object ResResource/ResLiteral $object)

boolean
contains
(obejct $resResource)

array
getMembers
()

boolean
isAlt
()

boolean
isBag
()

boolean
isSeq
()

void
remove
(obejct $object)

integer
size
()

Methods

Description |
Descendents |
Methods (details)

Constructor ResContainer (line 38)

Constructor You can supply a URI

- access: public

ResContainer

ResContainer
([string $uri = null])

- string
  $uri

add (line 50)

Add a new value to a container.

The new value is added as the last element of the container.

- access: public

void

add
(object ResResource/ResLiteral $object)

- object ResResource/ResLiteral
  $object

contains (line 68)

Determine whether the container contains a value

- access: public

boolean

contains
(obejct $resResource)

- obejct
  $resResource: ResResource/ResLiteral $resResource

getMembers (line 123)

Get an array of all resources that are values of this container

- access: public

array

getMembers
()

isAlt (line 90)

Returns true, if this resource is a container from type rdf:alt

- access: public

boolean

isAlt
()

isBag (line 101)

Returns true, if this resource is a container from type rdf:bag

- access: public

boolean

isBag
()

isSeq (line 112)

Returns true, if this resource is a container from type rdf:seq

- access: public

boolean

isSeq
()

remove (line 146)

Remove a value from the container.

Once removed, the values in the container with a higher ordinal value are renumbered. The renumbering algorithm depends on the type of container.

- access: public

void

remove
(obejct $object)

- obejct
  $object: ResResource/ResLiteral $resResource

size (line 181)

Returns the number values in the container.

- access: public

integer

size
()

#### Inherited Methods


Inherited From ResResource

> ResResource::ResResource()  
> ResResource::addProperty()  
> ResResource::equals()  
> ResResource::getAssociatedModel()  
> ResResource::getIsAnon()  
> ResResource::getProperty()  
> ResResource::hasProperty()  
> ResResource::listProperties()  
> ResResource::removeAll()  
> ResResource::removeProperties()  
> ResResource::setAssociatedModel()  
> ResResource::setIsAnon()  
> ResResource::setURI()

Inherited From Resource

> Resource::Resource()  
> Resource::equals()  
> Resource::getLabel()  
> Resource::getLocalName()  
> Resource::getNamespace()  
> Resource::getURI()  
> Resource::toString()

Inherited From Object

> Object::toString()

Documentation generated on Thu, 7 Jul 2005 13:43:31 +0200 by phpDocumentor 1.3.0RC3
